# Speciation of common Gram-negative pathogens using a highly multiplexed high resolution melt curve assay

**DOI:** 10.1038/s41598-017-18915-5

**Published:** 2018-01-18

**Authors:** Thomas Edwards, Shugo Sasaki, Christopher Williams, Glyn Hobbs, Nicholas A. Feasey, Katie Evans, Emily R. Adams

**Affiliations:** 1 Research Centre for Drugs and Diagnostics, Liverpool School of Tropical Medicine, Liverpool, UK; 2 School of Pharmacy and Biomolecular Sciences, Liverpool John Moores University, Liverpool, UK; 3 Malawi-Liverpool-Wellcome Trust Clinical Research Programme, Queen Elizabeth Central Hospital, Blantyre, Malawi; 4 Department of Clinical Sciences, Liverpool School of Tropical Medicine, Liverpool, UK

## Abstract

The identification of the bacterial species responsible for an infection remains an important step for the selection of antimicrobial therapy. Gram-negative bacteria are an important source of hospital and community acquired infections and frequently antimicrobial resistant. Speciation of bacteria is typically carried out by biochemical profiling of organisms isolated from clinical specimens, which is time consuming and delays the initiation of tailored treatment. Whilst molecular methods such as PCR have been used, they often struggle with the challenge of detecting and discriminating a wide range of targets. High resolution melt analysis is an end-point qPCR detection method that provides greater multiplexing capability than probe based methods. Here we report the design of a high resolution melt analysis assay for the identification of six common Gram-negative pathogens; *Escherichia coli*, *Klebsiella pneumoniae*, *Klebsiella oxytoca*, *Pseudomonas aeruginosa*, *Salmonella Sp*, and *Acinetobacter baumannii*, and a generic Gram-negative specific 16S rRNA control. The assay was evaluated using a well characterised collection of 113 clinically isolated Gram-negative bacteria. The agreement between the HRM assay and the reference test of PCR and sequencing was 98.2% (Kappa 0.96); the overall sensitivity and specificity of the assay was 97.1% (95% CI: 90.1–99.7%) and 100% (95% CI: 91.78–100%) respectively.

## Introduction

The determination of the species responsible for a bacterial infection is an important step for guiding patient management by tailoring antimicrobial therapy, and early identification is known to improve clinical outcomes^1^. The species identification of bacterial infections is equally valuable in a public health context, for detecting and tracking outbreaks of infection^2^. Gram-negative bacteria include many important human pathogens, and are known for their ability to develop extensive drug resistance. The Gram-negative members of the ESKAPE pathogens (*Klebsiella pneumoniae*, *Acinetobacter baumannii*, *Pseudomonas aeruginosa*, and *Enterobacter* species) are especially associated with nosocomial infections, and are frequently resistant to antimicrobial chemotherapy^3^ and therefore identification can trigger a change in treatment. Other notable Gram-negative pathogens include *Escherichia coli*, the leading cause of bacteraemia in the UK^4^, and serovars of *Salmonella enterica*, which are important causes of bloodstream infection (BSI) globally^5,6^.

Whilst the clinical presentation of these organisms may be very similar, optimal treatment often depends on the species in question. Frequently, used first line drugs for the empirical treatment of suspected bacterial infection such as the 3^rd^ generation cephalosporins and fluoroquinolones, are not effective against *P. aeruginosa*^7^. *A. baumannii* is frequently resistant to first line cephalosporins, such as ceftriaxone, and aminoglycosides^8^. Treatment of *Enterobacteriaceae* such as *E. coli* and *Klebsiella* is complicated by their ability to harbour a wide range of resistance genes, and required further testing to determine drug susceptibility.

Bacterial identification in clinical diagnostic laboratories in the UK is usually involves culture-based techniques, which can take between 8–120 hours depending on the sample type, organism and culture system used^9,10^. The isolate can then be classified as Gram negative or positive via microscopy of Gram’s stain, and identified via further culture on selective media and biochemical testing. This secondary culture step typically requires between 24 and 48 hours, and biochemical test panels such as the API 20E strips require 24 hour incubation^11^.

Molecular testing has been employed to identify Gram-negative bacterial isolates from primary cultures, and this approach can provide faster results than a second culture step. The implementation of rapid molecular testing has been shown to decrease time to optimal therapy^12^ and time to discharge^13^ in hospital settings.

Sequencing of 16S rRNA can be used for bacterial identification^14^, however is costly and time consuming, and therefore has limited clinical utility. Highly multiplexed commercial assays are available such as the FilmArray Blood culture identification panel (Biofire Diagnostics, US) and the Verigene Gram-negative blood culture assay (Nanosphere, US)^15^ which can provide results within 2 hours of initial culture positivity, however these tests are expensive and require dedicated instrumentation. Strategies relying on in-house PCRs are available, but typically require time consuming downstream analysis such as restriction fragment length polymorphism analysis^16^ or microarray^17^ due to known difficulties in multiplexing sufficient species in real-time PCR systems.

High resolution melt (HRM) analysis is an end-point qPCR detection method that differentiates amplicons based on their melt profile, enables greater multiplexing capability than probe based methods and is less expensive as it utilises an intercalating dye rather than fluorescent probes. During the HRM step, the reaction is heated in 0.1 °C increments, whilst fluorescence is monitored by the qPCR system. Any amplicons present after the PCR cycles dissociate at their melt temperature, which releases the intercalating dye and causes a decrease in fluorescence. Amplicons with different melt temperatures can be differentiated in this way, meaning multiple primer sets can be included in the same reaction as long as their melt temperature is sufficiently different, ideally by >1 °C.

HRM has been applied to the detection of bacterial resistance genes^18^, speciation of bacteria via 16S analysis^19^ and the detection of nosocomial pathogens^20^, however this technology has not been applied to the identification of multiple Gram-negative pathogens.

In this report, we have designed and performed a small scale evaluation of a HRM assay for detecting the Gram-negative pathogens *E. coli*, *K. pneumoniae*, *Klebsiella oxytoca*, *A. baumannii*, *P. aeruginosa* and *Salmonella* sp., combined with a Gram-negative specific 16S rRNA control, within a single reaction. *E coli*, *Klebsiella* and *P. aeruginosa* are the most common Gram-negative causes of blood stream infections^21,22^, whilst *A. baumannii* and *Salmonella enterica* infections are associated with a high mortality rate and benefit from early detection^5,8^. This panel of bacterial species was chosen as they are difficult to differentiate, frequently drug resistant, and cause infections in both the hospital and community^23^.

## Results

### HRM speciation assay

**Figure 1:**
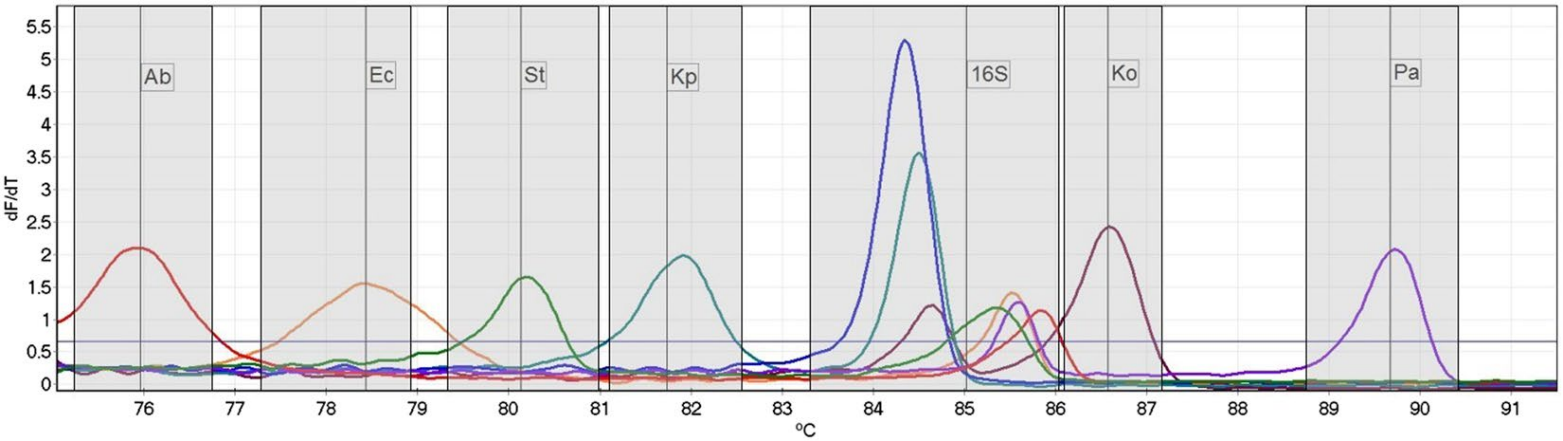
High resolution melt profile of the 6 amplicons for each species targeted by the assay, in addition to a single non-target Gram-negative isolate (*En. Cloacae*, dark blue line). All isolates have generated a 16S control peak over the calling threshold. Temperature ranges for result calling are indicated by the shaded areas, and the detection threshold indicated by the horizontal line. (Ab) *A. baumannii*, (Ec) *E. coli*, (S) *Salmonella*, (Kp) *K. pneumoniae*, (Ko) *K. oxytoca*, (Pa) *P. aeruginosa*.

When challenged with DNA from single isolates of each target species the multiplex HRM assay was able to differentiate all targets, with each peak separated by between 0.75 °C and 3.13 °C (mean 1.97 °C) from neighbouring peaks (Fig. 1). Each DNA sample also generated a peak for the 16S rRNA control, including DNA from an isolate of *E. cloacae*, a Gram-negative not included in the panel. The melt temperature (Tm) of the 16S rRNA peaks ranged over 1.5 °C (84.35 °C – 85.85 °C), due to the significant variation found in this region causing a variety of melt profiles.

### Limit of detection

The limit of detection, defined as the lowest concentration at which the bacteria were identified in all replicates, was between 1.6 × 10^3^ and 8 × 10^3^ CFU/ml for all the targets tested. Assuming 100% efficiency during the extraction process, this equates to between 4 and 20 genomes per PCR reaction.

### Assay evaluation

The HRM assay correctly identified 111 of 113 isolates (98.2% agreement, Kappa 0.96), including 68 out of 70 isolates of the target species (Table 1).

**Table 1:** Results of the HRM speciation assay tested on 113 Gram-negative bacterial isolates compared with a reference standard of 16S rRNA PCR and sequencing, by individual target.

HRM	Reference test - 16S PCR and sequencing								total
	Ab	Ec	S	Kp	Ko	Pa	16S	Negative	
*A. baumannii*	**1**	0	0	0	0	0	0	0	1
*E. coli*	0	**37**	0	0	0	0	0	0	37
*Salmonella sp*.	0	0	**1**	0	0	0	0	0	1
*K. pneumoniae*	0	0	0	**25**	0	0	0	0	25
*K. oxytoca*	0	0	0	0	**1**	0	0	0	2
*P. aeuruginosa*	0	0	0	0	0	**3**	0	0	3
16S	0	0	0	0	0	0	**99**	0	99
Negative	0	0	0	2	0	0	14	**608**	624
Total	1	37	1	27	1	3	113	609	791

Concordant results are highlighted. (Ab) *A. baumannii*, (Ec) *E. coli*, (S) *Salmonella*, (Kp) *K. pneumoniae*, (Ko) *K. oxytoca*, (Pa) *P. aeruginosa*.

**Figure 2:**
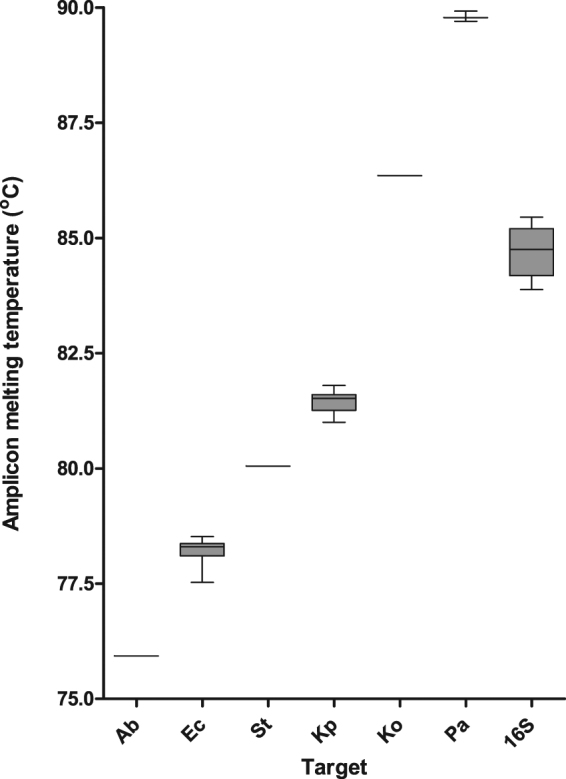
Variability of peak Tm of each target present in the collection of 113 isolates. (Ab) *A. baumannii*, (Ec) *E. coli*, (S) *Salmonella*, (Kp) *K. pneumoniae*, (Ko) *K. oxytoca*, (Pa) *P. aeruginosa*.

Overall the sensitivity and specificity of the assay were 97.1% (95% CI: 90.1–99.7%) and 100.0% (95% CI: 91.8–100.0%), respectively (Table 2). The peak Tm’s of for each species fell inside the predicted ranges without overlap between the different targets (Fig. 2).

**Table 2:** Accuracy of the HRM speciation assay, and of each primer set in the multiplex compared with the 16S rRNA PCR and sequencing.

Target	True positive	True Negative	False Positive	False Negative	Sensitivity	95% CI	Specificity	95% CI
overall	68	43	0	2	97.1%	90.1–99.7%	100%	91.78–100%
*A. baumannii*	1	112	0	0	100.0%	2.5–100.0%	100.0%	96.8–100.0%
*E. coli*	37	76	0	0	100.0%	90.5–100.0%	100.0%	95.3–100.0%
*Salmonella sp*.	1	112	0	0	100.0%	2.5–100.0%	100.0%	96.8–100.0%
*K. pneumoniae*	25	86	0	2	92.6%	75.7–99.1%	100.0%	95.8–100.0%
*K. oxytoca*	1	112	0	0	100.0%	2.5–100.0%	100.0%	96.8–99.9%
*P. aeuruginosa*	3	110	0	0	100.0%	29.2–100.0%	100.0%	96.7–100.0%
16S	99	0	0	14	87.6%	80.1–93.1%

The test failed to identify two of 27 *K. pneumoniae* isolates, which did not produce a peak with the required Tm but did produce a 16S rRNA peak, indicating that inhibition or DNA degradation were not the cause of the assay failure. No peaks outside the ranges of the calling bins were produced. All 43 non-target Gram-negatives were correctly classified by the assay, with only the Gram-negative specific 16S peak present in these isolates.

The 16S control was successful in 99 out of 113 isolates (86.7%), and in 43 out of 43 (100%) of the isolates not included in the panel.

### Mixed infection

The assay was able to detect both species in dual-bacterial cultures containing all combinations of the five species tested, with the exception of the *K. oxytoca* and *E. coli* mixed culture, in which only *E. coli* was identified (Table 3).

**Table 3:** The detection of mixed infections by the HRM speciation assay.

Targets	*A. baumannii*	*E. coli*	*K. pneumoniae*	*K. oxytoca*	*P. aeruginosa*
*P. aeruginosa*	2	2	2	2	
*K. oxytoca*	2	1	2		
*K. pneumoniae*	2	2			
*E. coli*	2				
*A. baumannii*					

Detection of both species in the mix is indicated by (2), detection of a single species indicated by (1).

## Discussion

Molecular species identification assays provide faster results than biochemical or culture based methods, enabling improved decision making^24,25^. The ability to deescalate to narrow spectrum antibiotics at an earlier point can benefit antimicrobial stewardship, safeguarding the effectiveness of current antibiotics^24^, and the identification of an organism likely to be resistant to first line antibiotics, such as *A. baumannii* or *P. aeruginosa*, enables targeted, personalised therapy.

The aim of this study was to develop a HRM based test for six common Gram-negative species, to provide rapid identification after the initial culture step and Gram staining. HRM enables greater multiplexing than expensive probe based qPCR assays, which are constrained by the number of optical detection channels, and is therefore well suited to assays with multiple targets. The HRM assay could detect seven targets, and the melt profile of each amplicon was highly specific, enabling automatic result-calling.

The assay uses individual primer sets for each target, generating a distinct peak that enables accurate speciation based on the peak Tm. An alternative strategy for HRM is to differentiate bacterial species using single or multiple sets of pan-species 16S rRNA primers, which can pick up a wide panel of species, but typically require significant data interpretation, such as the construction of multiple difference curves^19,26^. The use of separate primer sets allows for detection bins for automatic result calling, which provides unambiguous results and greatly simplifies the interpretation of the test, which is an important aspect when considering its use as a diagnostic.

The targets of the assay were selected as common Gram-negative pathogens able to cause community and hospital acquired outbreaks^23,27^. A well characterised Gram-negative specific control assay^28^ was included, to confirm the presence of a Gram-negative organism not included in the speciation panel. A negative result therefore would be interpreted as either a reaction failure or an incorrectly interpreted Gram stain. The test provided results within ~3 hours from a Gram-stained isolate, including the DNA extraction using a spin column based kit, which is comparatively faster than the approximately ~24 hours required for subculture^29^.

In the pilot evaluation, no false positive results were obtained for any target in the 113 isolates tested. This high specificity is important as decisions based on the identification of the causative species have the potential to alter patient management, and false positive results could lead to the selection of ineffective therapy. Overall the assay showed good agreement with the reference test, with a sensitivity and specificity of 97.1% and 100.0% respectively. Two *K. pneumoniae* isolates were not identified by the HRM assay. Recent studies on the genomic diversity of *K. pneumoniae* have suggested it should be split into 3 species; KpI, the most frequently encountered in humans, and KpII and KpIII^30^. BLASTn analysis of the primers used in the HRM assay showed mismatches to the *Khe* sequences of KpII and KpIII (GenBank Acssession numbers CP014156.1 and CP012252.1). It is possible the two isolates not detected fall into these groups, as the sequencing of the 16S rRNA that was carried out to characterise the strains does not discriminate between the different *K. pneumoniae* subtypes.

The ability of the assay to detect mixed Gram-negative infections is important as coinfections with multiple Gram-negatives do occur^31^ and polymicrobial samples constitute 2–12% of all positive blood cultures^15^. Current rapid systems for bacterial identification have poor sensitivity for detecting all species in mixed cultures^15,32^, which could lead to incorrect treatment. The HRM assay shows the potential to detect mixed cultures, however further validation is required with polymicrobial clinical samples.

The 16S control performed well in isolates not included in the panel, however failed to amplify in 14/68 isolates included in the panel. The failure of the 16S assay in these isolates is likely due to competition between the two amplicons for reagents during the reaction, such as dNTPs and Evagreen dye. The failure of the control assay in these samples would not impact on result interpretation, as the presence of a species-specific peak indicates successful amplification and a lack of sample inhibition.

The assay has been designed for use on DNA extracted from the first cultured isolate from a clinical sample, which usually contain high concentrations of good quality DNA, and we did not attempt to use the assay directly from clinical samples, such as urine or blood. The limit of detection of the assay ranged between 1.6 × 10^3^ and 8 × 10^3^ CFU/ml for the various target species, which is similar to that reported for other molecular assays for bacterial detection^33,34^. However, this indicates that some form of sample enrichment would be necessary for detecting bacteraemia, which can present with organism loads as low as 1 CFU/ml^10^. Further wok will be done to investigate the use of the test from clinical samples.

One limitation of this pilot study was the small numbers of available isolates of *P. aeruginosa*, *A. baumannii*, *K. oxytoca* and *Salmonella*, leading to large confidence intervals on the estimated accuracy of these components of the assay. A limitation of the assay as a molecular test is the inability to quantify the species via a standard curve. This is due to the non-specific nature of an intercalating dye meaning that any primer set, including the control included in every reaction, can generate fluorescence, making quantification unreliable as multiple targets may be amplifying simultaneously.

It is anticipated that this test could be a useful epidemiological tool for monitoring outbreaks and infections caused by the organisms included in the panel, with the advantage of more rapid results than culture or biochemical based tests, and a lower cost per assay than probe based qPCR. The use of the assay in this manner would first require the validation of the assay on a larger number of samples to more accurately determine its performance. Additionally, the test could be used to guide clinical management; an implementation study would be necessary to measure any improvement in treatment success or time to appropriate treatment resulting from the rapid detection of these organisms.

## Methods

### Bacterial isolates

A total of 111 bacterial isolates were obtained from a collection of clinical isolates sourced in the UK between 2012 and 2017 UK^35,36^. The isolates included *E. coli* (n = 37), *K. pneumoniae* (n = 27), *Enterobacter aerogenes* (n = 12), *Enterobacter cloacae* (n = 15), *Citrobacter freundii* (n = 12), *P. aeruginosa* (n = 3), *Morganella morganii* (n = 3), *K. oxytoca* (n = 1) and *A. haemolyticus* (n = 1). Additional DNA samples from *A. baumannii* (n = 1) and S*. enterica* serovar Typhi (n = 1) were received from Queen Elizabeth Hospital, Blantyre, Malawi. The use of the Malawian isolates was approved by the University of Malawi College of Medicine Research and Ethics Committee (COMREC), Blantyre, under study number (P.08/14/1614). The handling and culture of isolates was carried out under biological safety category 2 conditions, including the use of a Class II safety cabinet.

Isolates were identified by sequencing amplicons of a 16S rRNA PCR performed using the 27 F and 1492r primers according to a published protocol^37^. Sanger sequencing was carried out commercially (Source Bioscience, UK) and sequence data was analysed using BLAST (https://blast.ncbi.nlm.nih.gov).

### DNA extraction

DNA was extracted from single distinct colonies of the isolates after 24 hours growth at 35 °C on Luria-bertani (LB) agar, using a DNeasy kit (Qiagen, Germany) following the protocol for extraction from Gram-negative bacterial culture. DNA was eluted into 200 µl of elution buffer, and stored at −20 °C until use.

### Primer design

Specific primer pairs were designed for four of the seven targets of the assay. Primers for *Salmonella* sp.^38^, *P. aeruginosa*^39^ and the Gram-negative 16S control^28^ were taken from previously published assays due to their good performance in initial tests. Candidate target genes for each organism were identified from previous studies^40–43^, and all available gene sequences for each target were downloaded from GenBank. The sequences were aligned using MEGA 7^44^ and primers were designed for conserved sites using Primer3 (http://primer3.ut.ee/). Each primer set was designed to produce an amplicon with a distinct peak melting temperature (Tm) to enable their discrimination during HRM analysis. OligoCalc nearest neighbour method was used to estimate amplicon melt temperatures based on the amplicon sequence (http://biotools.nubic.northwestern.edu/OligoCalc.html). Primer specificity was tested using BLAST, with particular focus on genetically similar Gram-negative strains. Primer sequences, their targets, and predicted amplicon Tm are shown in Table 4.

**Table 4:** Primer sequences, target genes, amplicon sizes and predicted Tm for the assays.

Organism	Target gene	Forward primer sequence (5′-3′)	Reverse primer sequence (5′-3′)	Concentration (nM)	Amplicon size (bp)	Predicted Tm (°C)	Citation
*Acinetobacter baumannii*	16S *rRNA*	CCCACCATGACTTTGACTGG	GGCGCTCTACCAACTAAGCT	100	91	76.41	this study
*Escherichia coli*	*uidA*	TCTGGCAACCGGGTGAAG	TAGATATCACACTCTGTCTGGCT	400	73	77.07	this study
*Salmonella sp*.	*invA*	AGCGTACTGGAAAGGGAAAG	CACCGAAATACCGCCAATAAAG	80	123	80	^38^
*Klebsiella pneumoniae*	*Khe*	CATCTGCCACACCTTTCTCA	CCGGGATTGAGCGGGTAATA	400	105	81.12	this study
Gram-negative control	16S *rRNA*	AYGACGTCAAGTCMTCATGG	AGGAGGTGATCCAACCGCA	400	353	85.65	^28^
*Klebsiella oxytoca*	*pehX*	TACCGTCACGCACTATCCTC	TCAAGCGGATACTGGGCC	400	153	86.04	this study
*Pseudomonas aeruginosa*	*OatA*	CTGGGTCGAAAGGTGGTTGTTATC	GCGGCTGGTGCGGCTGAGTC	100	232	89.02	^39^

### HRM speciation assay

Each 12.5 µl reaction of the HRM speciation assay included 6.25 µl of Type-it 2 × HRM buffer (Qiagen, Germany), 400 nM of each forward and reverse primers targeting *E. coli*, *K. pneumoniae*, and *K. oxytoca*, 100 nM of each primer for *A. baumannii*, and *P. aeruginosa*, and 80 nM of each primer for *Salmonella* sp. Forward and reverse primers targeting a generic bacterial 16S rRNA sequence u were present at an 80 nM concentration. Molecular grade water was then added to a final reaction volume of 12.5 µl, including 2.5 µl of DNA template. Reactions were thermally cycled in a RGQ 6000 (Qiagen), with the following thermal profile: Taq activation at 95 °C for 5 minutes, followed by 40 cycles of 95 °C for 10 seconds, 55 °C for 30 seconds, 72 °C for 10 seconds. Following this, HRM was carried out by melting from 72 °C to 92 °C, taking a reading in the HRM channel every 0.1 °C, with a 2 second stabilisation between each step. Data was visualised as the negative first derivative of the melting curve to show peak fluorescence dissociation. Positivity was indicated by a peak at the predictive Tm of the target, above the set cut off value. All analysis was carried out using the RGQ system software.

The assay was initially tested using a single isolate for each target, and a threshold value for peak calling was set at 0.525 dF/dT (10% max dF/dT obtained) and retained for all future experiments. To allow automatic result calling, calling bins were stipulated in the RGQ software using this data. Calling bin widths of 1.6 °C for target identification were also set 0.8 °C above and below the peak Tm for the *A. baumannii*, *E. coli*, *S*. Typhi, *K. pneumoniae* and *P. aeruginosa*. To account for the greater spread of the 16S peak Tm’s, a larger bin of 2.7 °C was set from 0.2 °C above the highest 16S Tm obtained, and 1 °C below the lowest. The *K. oxytoca* bin was reduced to 0.5 °C above and below the peak Tm in order to account for close proximity to the wide range of the 16S peak. The calling bins were set as follows; *A. baumanii* 75.16–76.76 °C, *E. coli* 77.29–78.89 °C, *Salmonella sp*. 79.34–80.94 °C, *K. pneumoniae* 80.97–82.57 °C, 16S 83.35–86.05 °C, *K. oxytoca* 86.07–87.07 °C and *P. aeruginosa* 88.87–90.47 °C.

### Limit of detection

The limit of detection (LOD) of the assay was determined for each of the target species. A single colony of each bacteria was added to 5 ml of LB media, homogenised by shaking, and then incubated at 37 °C for three hours. The cultures were then sequentially diluted 1:10 in LB media, and 10 µl of each dilution was plated in triplicate on LB agar. The plates were then incubated overnight at 37 °C and the colonies counted to quantify the CFU/ml in the suspension. After the suspensions were plated, 200 µl aliquots were taken from each one and the DNA was extracted using a DNeasy kit (Qiagen), following the manufacturer’s instructions. Each DNA sample was tested using the HRM speciation assay in triplicate, to determine the LOD. The LOD was defines as the lowest concentration of bacteria that generated a positive result in all triplicates. *S. typhi* was only available to us as a nucleic acid sample, and so could not be included in this experiment.

### Pilot evaluation

The assay was evaluated by testing randomised DNA samples from the 113 Gram-negative isolates. Positivity calling bins and the detection threshold were automatically set from optimisation experiments with control isolates. DNA samples were tested once, with the result automatically scored by the RGQ data analysis software. The samples used to initially develop the test were randomised and added back to the sample collection prior to the evaluation due to low numbers of *Salmonella* sp. and *k. oxytoca*. The results were then compared with the results of the 16S rRNA sequencing to estimate sensitivity and specificity.

### Mixed infections

To determine whether the assay could identify mixed infections, mixed cultures were grown on LB agar to simulate culture from a mixed clinical sample. Single colonies of *P. aeruginosa*, *K. pneumoniae*, *K. oxytoca*, *A. baumanii* and *E. coli* were streaked onto LB agar as either two, three or four species mixed cultures, in every combination*. S. typhi* again was not included due to a lack of available isolate.

A loop of bacteria was taken from an area of the plate that appeared to have mixed growth. If no mixed growth could be detected by eye the loop was taken from the area of heaviest growth on the plate. DNA was extracted as described, and then tested using the speciation HRM assay.

### Data availability

All data generated during this study is presented in an analysed format is this manuscript. Raw datasets generated during the current study are available from the corresponding author on reasonable request.
